# The interplay and incremental development of tidal stream arrays in the Pentland Firth

**DOI:** 10.1007/s40722-026-00490-5

**Published:** 2026-04-15

**Authors:** Misha D. Patel, Amanda S. M. Smyth, Athanasios Angeloudis, Thomas A. A. Adcock

**Affiliations:** 1 Department of Engineering Science, University of Oxford, Parks Road, Oxford, OX13PJ UK; 2 School of Engineering, University of Edinburgh, West Mains Road, Edinburgh, EH93JG UK

**Keywords:** Tidal stream energy, Tidal turbine arrays, Pentland Firth, Incremental development, Regional modelling, Tidal array interactions

## Abstract

The development of tidal stream energy sites is constrained by numerous practical, technical, and accessible constraints, including changes in the flow caused by the presence of the tidal farm. Large and complex sites are typically developed incrementally and may involve multiple developers. This regional modelling case study of the Pentland Firth, widely regarded as one of the most significant global locations for tidal energy extraction, investigates these dynamics. This study examines scenarios for the incremental development of the Pentland Firth, incorporating assumptions regarding tidal farm design. The analysis considers a range of configurations, including variations in turbine density and the incorporation of shipping lanes within designated lease areas. The study finds that there are interactions between individually developed tidal farms of the site, but they are moderate with the power in a given farm unlikely to vary by more than 20% (based on a positive interaction), as a result of the development of other farms in other parts of the Pentland Firth. To minimise such array interactions, it is recommended that site leasing be based on the allowable thrust applied to the flow rather than on projected power generation. Furthermore, the findings suggest that the maximum power generated from turbines in the Pentland Firth, averaged over time, is unlikely to exceed approximately 1 GW, broadly consistent with most estimates in the literature.

## Introduction

The Pentland Firth is widely considered to be the most important site for tidal stream energy in the world (Coles et al. 2025). Whilst the ambiguity in the magnitude of the resource is central to the present paper, many assessments suggest that over half of the UK’s tidal resource is in the strait, and in Europe, only the tidal race around Alderney likely has the same order of magnitude of resource (DTI 2008). Determining the scale of this resource is essential for the tidal stream industry and for policymakers evaluating tidal stream power’s potential contribution to the UK energy mix. Quantifying its magnitude is also vital for informing investment decisions and assessing its role in reducing dependence on finite fossil fuels.

A key issue, however, is that site-based resource assessments can vary by orders of magnitude, with national and global resource assessments being equally variable. This is partly due to the complexity of tidal flow fluid mechanics and turbine-flow interactions, but also due to the consideration of constraints limiting the viable extraction of the resource. There is a hierarchy of different approaches which can be taken (Patel et al. 2023) but resource estimates at present require significant assumptions to be made.

Cartwright et al. (1436) estimate the total power from tidal waves coming in to the UK from the continental shelf is approximately 250 GW, but only a fraction of this can be extracted (MacKay 2016). The technical potential of the resource has been estimated to be up to 22.5 GW (Hm Government 2010), although this amount of power could not be extracted. A number of studies assess the UK’s average tidal stream resource to be 1.5–22.5 GW (Black and Veatch 2005; Metoc 2007; Carbon Trust 2011). Carbon Trust (2011) states that re-prioritising the use of offshore sites could increase their assessment of the practical resource to 3.3 GW. Site-based assessments are also wide-ranging due to the use of different models and assumptions, which prevents them from being used as part of an overall UK resource assessment.

Resource assessments can assist with turbine design by providing data to characterise hydrodynamic effects, identifying appropriate sites for tidal stream developments and assessing the overall production of energy from an array over the lifetime of a project (Thiébot et al. 2022). Therefore, a refined resource assessment is necessary to inform investment decisions and for the development of technology and array implementations to continue.

A related problem is how tidal sites should be developed. Given the number of tidal sites is limited it is important that key sites should be developed as efficiently as possible. However, there is a significant interaction between tidal turbines and the tidal flow. Hence, placing a tidal farm in one area of a site will lead to the flow changing elsewhere.

**Fig. 1:**
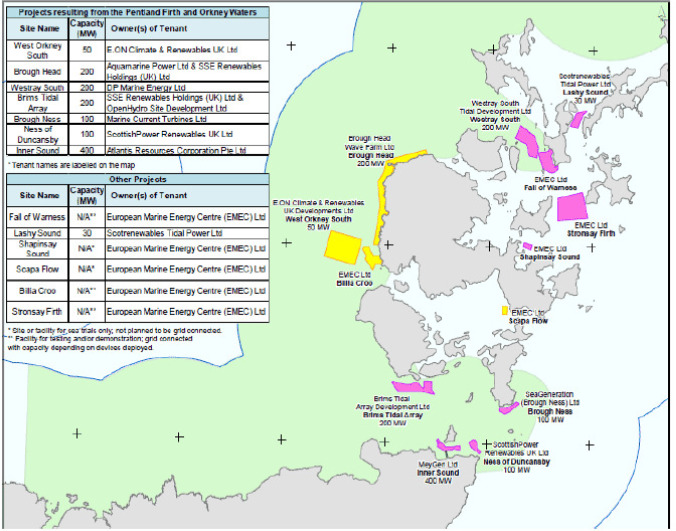
Planned lease sites for wave (yellow) and tidal stream (pink) developments in the Pentland Firth and Orkney in 2010. Source: Scottish Government (2018)

Whilst noting that these may change going forward, in 2010 four lease sites were agreed by the Crown Estate and leased to four different developers (The Crown Estate 2023). Figure 1 presents planned lease sites in the Pentland Firth and Orkney for wave and tidal stream developments in 2010. Of the four original lease sites in the Pentland Firth, three were discontinued after 2015, leaving the Meygen site in the Inner Sound as the only site being actively developed, with Phase 1 already operational (Meygen 2012). The Ness of Duncansby lease has been reconsidered but has not progressed beyond the pre-planing stage. The original Crown Estate lease sites were outlined based on less-refined, older modelling and have the potential to be updated as resource assessments are refined and seabed usage is re-prioritised. The study undertaken by Goss et al. (2018) highlights the need to carefully consider the development of independent arrays in order to maximise resource extraction for each developer.

The aim of the present paper is to look at one approach for how the Pentland Firth could be developed through multiple lease sites, and considers the interactions between these sites. This approach can inform the The Crown Estate and The Crown Estate Scotland ’s definition of lease sites, enabling independent developers to develop sites individually, whilst minimising negative interference. This is crucial because the original lease sites in the Pentland Firth were leased to different developers, therefore, developments needs to be considered in a way that minimises negative interfering effects from isolated development. In addition, considering this problem contributes to an understanding of the tidal stream resource of the site.

The approach taken in this study is to consider a plausible scenario for how a site may in practice be developed. This requires substantial assumptions to be made, and whilst such an approach cannot give a definitive value of the resource, it is suitable in guiding developers and policy makers on the magnitude of resource.

## Background

### Tidal stream resource assessment

Resource assessments of tidal stream energy have been the subject of a number of studies, employing a range of methods to model site hydrodynamics and extraction of energy through different representations of turbines. Accurately quantifying the resource of tidal stream energy requires an understanding of the interactions between turbines and the flow across various scales inherent in tidal energy investigations. To manage the complexity of this multi-scale problem, the scales are often approximated as decoupled, resulting in a number of simplifying assumptions and necessary compromises.

Analytical and numerical models can be used to assess the resource of tidal stream energy. Predicting power available to a turbine as a portion of the kinetic energy flux has been used in the wind industry and early attempts to quantify tidal stream energy resource have adopted this approach (Fraenkel 2002; Black and Veatch 2005; Blunden and Bahaj 2007). However, the method does not consider the presence of turbines and their impact on the flow, nor does it consider the configuration of closely arranged turbines in rows, which can theoretically extract more power than the undisturbed kinetic energy in the channel (Vennell 2013; Adcock et al. 2014). Garrett and Cummins (2005) demonstrated, through a simplified model, that the overall drag coefficient of a channel increases when turbines extract power, slowing down the flow in the channel. Acknowledging the impact of turbines on the resource, Black and Veatch (2005) introduced a ‘significant impact factor’(SIF) to calculate extractable power without significant environmental and economic impact. Blunden and Bahaj (2006) produced a time-series of tidal velocity data at Portland Bill and gave an example of how to use the data to predict power outputs by assuming the power as a fraction of kinetic energy. However, Garrett and Cummins (2005) demonstrated that the effect of tidal stream turbines on the flow is not directly related to the undisturbed kinetic flux.

To date, all resource assessments of real sites have had to be made using numerical models due to the limitations of experiments (see for instance Draper et al. (2013)). Models of the complex multiscale problem of energy extraction are challenging and this remains a considerable source of uncertainty. Until large tidal farms are developed, full validation of resource models will not be possible.

An effective approach is to apply an upper-bound constraint. This can either be done by finding the optimal resistance, which maximises power extraction (Garrett and Cummins 2005), or using an idealised turbine representation and adding turbines until the power per swept area falls below a reference value (Adcock et al. 2013). However, these are explicitly upper bounds and not technically achievable.

### Tidal stream resource of the Pentland Firth

The Pentland Firth, one of the most notable sites for tidal stream energy in the UK, is a strait between the north coast of Scotland’s mainland and the Orkney Islands. The strong currents in the area are due to the elevation phase difference between the east and west of the Pentland Firth, which is enhanced by tidal streaming due the area’s topography (Neill et al. 2017). The area is divided into multiple streams due to the presence of two islands, Stroma and Swona. A number of studies have been conducted on the tidal stream energy resource at the Pentland Firth, with assessments ranging from 1 to 17.7 GW (Black and Veatch 2005; Salter and Taylor 2007; Easton et al. 2012; Adcock et al. 2013; Draper et al. 2014b; O’Hara Murray and Gallego 2017; De Dominicis et al. 2017; Wang and Adcock 2018; Jordan et al. 2022). The large range highlights the inconsistency in resource assessments and the need for a refined method to quantify the resource.

Black and Veatch (2005) applied the kinetic flux method to quantify tidal stream energy in the Pentland Firth. Salter and Taylor (2007) suggest that studies based on the kinetic flux under-predict the Pentland Firth’s resource. Salter (2009) further argued that estimates based on open flow field equations, used for predicting the output of wind turbines, and incorrect turbine design can lead to the potential resource of tidal stream energy to be underestimated by up to two orders of magnitude. Salter and Taylor (2007) used the electrical circuit analogy to describe channel impedance in the Pentland Firth. Their assessment of 17.7 GW at peak flow was based on the assumption that energy lost to bed friction will be similar to the energy extracted by turbines. Salter (2009) highlights the uncertainty of bed friction losses in the Pentland Firth. The method is highly dependent on the value of bed friction and the uncertainty in the parameter impacts the overall assessment greatly. Salter (2009) assumes that flux in the channel is unchanged by the presence of turbines. However, the current changes when resistance is added to the flow, which is a significant omission from Salter ’s method and indicates why the assessment is much greater in comparison to other assessments discussed. The assessment by Salter and Taylor (2007) is based on peak flow, therefore the power averaged over the tidal cycle will be significantly lower.

Carbon Trust (2011) assessed the practical resource potential of the Pentland Firth as part of a national study, using a depth-averaged SWE model. They revised an initial estimate of 0.9 GW by relaxing the constraints based on environmental and economic considerations, recognising the cost-benefits of economies of volume for large scale development at the Pentland Firth and acknowledging the fact that the model did not accurately represent environmental impacts. The updated assessment increased by 120%, highlighting the sensitivity of resource assessments to assumptions on limitations to the resource (Coles et al. 2021).

Easton et al. (2012) calculated the mean energy flux into the Pentland Firth but did not consider the practically extractable resource, stating that it was unlikely a maximum of 10 GW flux could be extracted. Meteorological forcing and additional flow sources were excluded from the study. Only the astronomical and Coriolis forcing were incorporated, as they were considered to be more significant. Easton et al. (2012) noted the limited availability of in-situ data for modelling. Numerical models are useful, however, lack of comparable field data makes verifying results difficult. This highlights a significant challenge of carrying out a validated and accurate resource assessment.

Adcock et al. (2013) assessed the upper bound power available to three rows of turbines occupying the width of the Pentland Firth. They found that for a given number of rows, the power available is greater with a larger blockage but as more rows are added, the increase in power shows diminishing returns. Therefore, a minimum incremental power per swept area of 1 kW/m$^2$ was implemented as a crude equivalent to offshore wind. They suggest a refined assessment will unlikely exceed their upper bound assessment of 1.9 GW because the model overestimates power available compared to a turbine that will generate an equivalent opposing force on the flow. An assumption of the model is that the maximum reduction in peak flow rate at the site must be no greater than 30%, which is likely to exceed environmental impact restrictions. It is unlikely that a row of turbines would be deployed across the entire width of the Firth due to other seabed usage priorities, such as shipping channels.

Draper et al. (2014b) assessed potential power from sub-channels in the Pentland Firth to be up to 4.19 GW, highlighting that it cannot be described by a single value. They acknowledge the power potential of channels are dependent on the deployment and operation of arrays in parallel or series channels. They found that developments of parallel sites increased power extraction but sites developed in series experienced losses in power extraction. This highlights a risk of sites being developed in isolation when leased to different developers. Their assessment does not consider power available to turbines.

O’Hara Murray and Gallego (2017) applied a 3-D model to the Pentland Firth. The model assumes turbine performance is the same regardless of flow direction. This would require a yawing mechanism and could have an impact on flow dynamics near turbines. The assessment is a theoretical upper bound, not a viable resource assessment because the power extraction refers to power removed from the flow due to turbines exerting thrust on the flow rather than electrical power. Their assessment requires a large array (up to 31,420 turbines) to be deployed across the entire width of the Pentland Firth, causing a 38% volume transport reduction and changes up to 2 ms$^{-1}$ in flow velocity. The scenario they modelled would likely exceed environmental and leasing regulations. The study does also present a more realistic scenario (5636 turbines) with less environmental impact, reducing the assessment by 72%, to 1.4 GW.

De Dominicis et al. (2017) used a 3D model to assess the ocean’s response to energy extraction from a large array, environmental impact and power available for electricity generation. They estimate the resource to be 1.64 GW, which is of the same order of magnitude as that assessed by O’Hara Murray and Gallego (2017) (moderate environmental impact scenario). This site-based study does consider lease site area restrictions making it a more refined assessment.

### Deployment of multiple arrays at a site

Goss et al. (2018) investigated two contiguous arrays in the Alderney Race, developed by Alderney and France. They explored scenarios of collaborative, successive and independent developments. The study focussed on the micro-siting of turbines and the competition effects between the arrays depending on the development strategy. The greatest power output is achieved when the arrays are developed collaboratively, although the power output between each scenario is similar. The array developed by France is found to have a greater impact on the array developed by Alderney and that a co-operative development would be more beneficial to Alderney. The effect of array interactions and consequences for independent developers are also highlighted by Waldman et al. (2019) in the context of policy implications.

Another study that investigates the interaction of multiple tidal stream developments in the Channel Islands was undertaken by Coles et al. (2017), looking at the Alderney Race in conjunction with two other potential sites, the Casquets and Big Roussell. The upper bound estimate of the resource suggests the majority of the resource can be extracted in the Alderney Race. When power is extracted in the Alderney Race, the head difference and volume flux through the Casquets, a parallel site located 3 km away, increases as the flow increases around Alderney. This leads to constructive effects for power extracted in Casquets, when developed together with other sites. The third site, Big Roussel is located 40 km downstream of the Alderney Race and Casquets and experiences a reduction in extracted power when developed with the other sites. They find that during the ebb tide, the flow diverts around the Channel Islands when power is extracted in the Alderney Race and Casquets, leading to a volume flux reduction through Big Roussel compared to when power is extracted through Big Roussel alone. The study highlights the interaction between sites is highly dependent on their location relative to each other.

The interaction between four tidal arrays in the Pentland Firth is considered by Funke et al. (2016), through the implementation of a gradient-based optimisation method. Three of the arrays coincide with the Inner Sound, Ness of Duncansby and Brough Ness lease sites in Fig. 1, and the fourth array is placed in Cantick Head, further east of the Brims tidal array development. They demonstrate that optimising the arrays individually and simultaneously leads to different outcomes for the resource. When the four sites are optimised individually the potential of each farm is overestimated. The Cantick Head and Brough Ness arrays have minimal differences when optimised individually or simultaneously because they are far enough apart that interference is minimised. However, the Inner Sound and Ness of Duncansby arrays lie directly behind each other in the direction of the flow and are in relatively close proximity. The optimisation of the Ness of Duncansby array is notably affected by the presence of the Inner Sound array, as the latter experiences more constrained flow due to the presence of Stroma. The present paper considers more incrementally how the overall Pentland Firth site will be developed considering other factors such as the change to the flow.

As the tidal energy sector continues to evolve, the deployment of multiple arrays in close proximity becomes increasingly common, leading to interactions between them. The studies outlined in this section have highlighted the importance of considering these effects in the development of sites. Other than studies that consider lease sites, the identification of the multiple arrays has not been strategic and very few studies consider the practical constraints on the resource. In this study, the development of multiple tidal stream arrays across the Pentland Firth, with the consideration of practical constraints on the resource, is investigated to assess and refine the assessment of the tidal stream resource in the Pentland Firth and inform the strategic development of the site.

## Model

### The *Thetis* shallow water equation model

The 2D shallow water equations are the standard equations used in flow models of tidal energy sites (Blunden and Bahaj 2006; O’Rourke et al. 2010; Serhadlıoğlu et al. 2013). In this study, *Thetis* is used, which employs an unstructured grid and *Firedrake*’s finite element partial differential equation solver (Kärnä et al. 2018; Rathgeber et al. 2016). The non-conservative form of the non-linear shallow water equations are solved in *Thetis* (Eqs. 1 and 2) (Opentidalfarm 2016; Kärnä et al. 2018; Goss et al. 2020);

1 $$\begin{aligned} &  \frac{\partial \eta }{\partial t}+\nabla \cdot (H \textbf{u})=0, \end{aligned}$$

2 $$\begin{aligned} &  \frac{\partial \textbf{u}}{\partial t}+\textbf{u} \cdot \nabla \textbf{u}-\nu \nabla ^2 \textbf{u}+f \textbf{u}^{\perp }+g \nabla \eta \nonumber \\ &  \quad =-\frac{\tau _b}{\rho H} -\frac{c_t}{\rho H}||\textbf{u}||\textbf{u}, \end{aligned}$$

where $\eta$ is the free surface elevation, $t$ is time, $H$ is the sum of the free surface elevation and depth at rest (i.e. total water depth), the vector $\textbf{u}$ represents the components of depth-averaged velocity, $\nu$ is kinematic viscosity, $f \textbf{u}^{\perp }$ represents the force of the Coriolis effect, $g$ is acceleration due to gravity, $\tau _b$ is the bed friction and $\rho$ is the water density (Kärnä et al. 2018; Opentidalfarm 2016; Goss et al. 2020). The term in Eq. 2 with drag coefficient, $c_t$, is the sink term that represents turbines and their support structures in an array and can be related to the turbine’s thrust coefficient, $C_T$, swept area of the turbine, $A_T$ support structure drag, $C_{D,S}$, support structure area, $A_T$, and the turbine density, $d({\textbf {x}})$, as $c_t(d({\textbf {x}}))=(\frac{1}{2}C_T({\textbf {u}}({\textbf {x}})) A_T + C_{D,S}A_{S}) d({\textbf {x}})$ (Funke et al. 2016).

Equation 3 approximates the rotor thrust force exerted by ‘$N$’ number of turbines in an array, where $C_T$ is the thrust coefficient and $A_T$ is the swept area of an individual turbine rotor,

3 $$\begin{aligned} {F}_\textrm{array}=\sum _{i=1}^N \frac{1}{2} \rho C_T A_T\left\| \textbf{u}_i\right\| \textbf{u}_i. \end{aligned}$$

The free stream velocity acting on the $i{\text {th}}$ turbine is represented by $\textbf{u}_i$. The thrust coefficient is dependent on the velocity according to a performance curve and individual turbine rotor area in the array, described in Eq. 3, is assumed to be homogeneous. The instantaneous power produced by the array is given by Eq. 4, where, $c_p ({\textbf {x}})$ is the power coefficient function defined as, $c_p (x) = C_p(\textbf{u}({\textbf {x}}))A_T d({\textbf {x}})$.

4 $$\begin{aligned} P_\textrm{array} = \int _{\Omega _\textrm{array}}^{}\frac{1}{2}\rho c_p ({\textbf {x}})|\mathbf {{\textbf {u}}}({\textbf {x}})|^3 d{\textbf {x}}. \end{aligned}$$

Bathymetry data are taken from Edina Digimap and tidal constituent data from TPXO8-atlas (Edina Digimap Service 2020; Egbert and Erofeeva 2002). The mesh used 20,710 nodes, with a minimum element size of 60 m in the area where turbines are placed. The unstructured triangular mesh was created in *qmesh* (Avdis and Hill 2017; Avdis et al. 2018).

The model is forced with semi-diurnal lunar constituent, M$_2$, and semi-diurnal solar constituent, S$_2$, data for 14 days, representative of the spring-neap cycle, once fully evolved flow conditions were established from an initial state of equilibrium. Two constituents are sufficient to capture the key characteristics of the Pentland Firth tidal site (Adcock et al. 2014; Patel et al. 2023) and this study primarily considers the relative changes between cases.

### Turbine array representation

There is a fundamental challenge in modelling energy extraction from depth-averaged flow models as the real fluid mechanics is three-dimensional. One way the presence of turbines can be accounted for is by calculating the head loss across turbines as line discontinuities (Draper et al. 2010) or other sub-grid scale models (Djama et al. 2022). Altering the bed friction can also be used to model the presence of turbines (Sutherland et al. 2007; Yates et al. 2013).

Turbines are represented in *Thetis* as an additional sink term in the momentum equation, to include the force on the flow due to turbines (Opentidalfarm 2016; Kärnä et al. 2018; Jordan et al. 2022). There are two implementations of turbines in *Thetis*-continuous or discrete. In the discrete model, a ‘bump function’ is used to vary drag smoothly over the representative area of the individual turbine (Funke et al. 2014). This necessitates that the mesh resolution in turbine regions is finer than the turbine’s diameter. This technique is most effective for individual arrays with a limited number of turbines, as larger arrays make the approach computationally expensive, restricting the scope for parameter exploration in this study. Conversely, the continuous method distributes drag over the area representing the array (as employed in Kramer et al. (2015); Funke et al. (2016)). In the present study, the continuous approach is chosen, given the focus on assessing the site’s resource behaviour under varying turbine specifications in arrays, rather than the precise placement of individual turbines. As such, turbines are not individually resolved. Instead, a ‘turbine density’ is applied to the specified subdomain (Eq. 5).

5 $$\begin{aligned} {\text {Turbine density}} = \frac{{\text {Number of turbines}}}{{\text {Representative planform area}}}. \end{aligned}$$

The local blockage, which describes the proportion of the local passage cross-sectional area that a single device occupies, can be calculated using,

6 $$\begin{aligned} B_L=\frac{ \text{ single } \text{ device } \text{ area } }{ \text{ local } \text{ passage } \text{ cross-sectional } \text{ area } }=\frac{\frac{\pi d^2}{4}}{h(d+s)} \end{aligned}$$

where *d* is turbine diameter, and *s* the spacing (Nishino and Willden 2012). See Fig. 3 in Patel et al. (2024) for a schematic of turbine density and blockage.

**Fig. 2:**
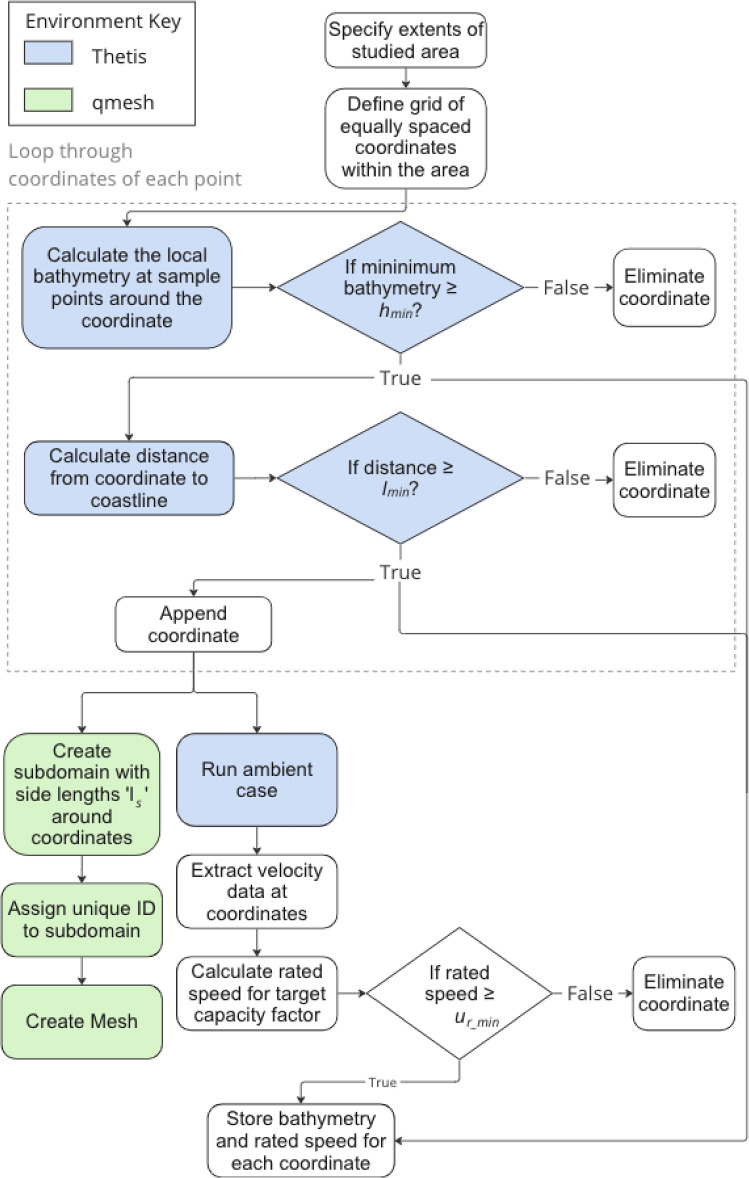
Method for creating the mesh and collecting data for assigning the turbine specifications in each array area. Source: Patel et al. (2024)

Accounting for local blockage is crucial in models, as it enhances the energy extraction potential of tidal turbines (Nishino and Willden 2012; Dehtyriov et al. 2021). The influence of both local and global blockage is represented in shallow water equation models and has been illustrated in an idealised channel by Chen et al. (2019a); Bonar et al. (2019). However, assessing the blockage experienced by turbines in practice is challenging due to the variability in depth and width of real-world channels, with blockage depending on the cross-sectional area of the flow. There is no perfect method for incorporating blockage effects into models, as different modelling approaches and assumptions influence how turbines and their interactions with flow are represented. In this study, blockage-corrected blade element momentum theory is employed to determine the power and thrust coefficients for the turbines included (Vogel et al. 2018; Chen 2019). It is assumed that performance curves of all turbines use variable-speed variable pitch (VSVP) control. VSVP control strategy is usually used for large diameter turbines, whilst smaller turbines typically operate with fixed pitch (Adcock et al. 2021). It is likely that developments of the key Pentland Firth site will include larger turbines, hence VSVP turbines are modelled.

**Fig. 3:**
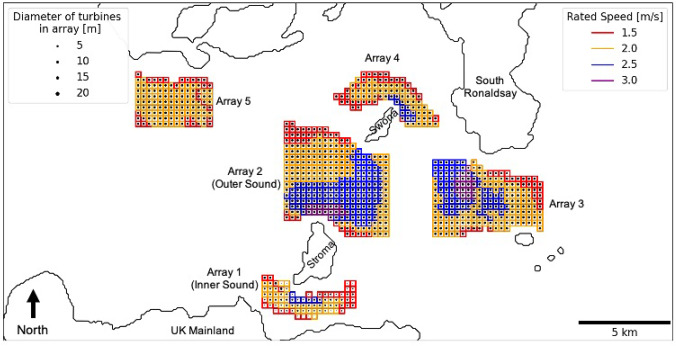
All arrays identified for deployment using the extended heterogeneous array design framework. The edge colour of squares represents the rated speed of turbines in each array area, whilst the size of the black circle indicates turbine diameter. Arrays are numbered in order of specification to illustrate the sequential development strategy across the Pentland Firth

## Development of the Pentland Firth

### Heterogeneous array design approach

The approach undertaken for identifying multiple arrays across a site, and assigning the turbine specification (i.e. turbine diameter and rated speed) is outlined and presented in Fig. 2 (Patel et al. 2024). The heterogeneous array design framework, developed in Patel et al. (2024), is applied across the Pentland Firth to identify additional areas to deploy arrays based on the concentration of the resource across the site. A grid of homogeneous sub-arrays are defined, so only one specification of turbines is present in that array. The turbine specification refers to the diameter and rated speed of the turbines and sixteen turbine specifications are considered; four rotor diameters (5, 10, 15, and 20 m) and four rated speeds (1.5, 2.0, 2.5, and 3 m/s). The framework defines the diameter and rated speed of the turbines based on bathymetry and flow field data. The application of the framework is fully described in Patel et al. (2024).

Successive arrays are defined using the flow field of the previous deployment strategy to account for effects of the increasing scale of development. The Inner Sound array modelled in Patel et al. (2024) is considered as the first array, given its pre-eminent status and the current developments which are going in this area. A minimum distance of 1.5 km between independent arrays is maintained, a similar distance between the original Inner Sound and Ness of Duncansby lease sites (Fig. 1).

### Turbine foundation parameterisation

A key issue to consider, in the study of the incremental development of the site, is the water depth and the impact this has on tidal turbine foundations. The Pentland Firth has depths up to 100 m, with an average of approximately 60 m across the area (Marine Scotland 2016). The Inner Sound is the shallowest area of the site with turbines being placed in depths no greater than 50 m. However, other areas across the site are characterised by deeper bathymetry. While most large diameter turbines are bottom-fixed and smaller turbines are deemed more suitable for floating foundations, as detailed by Adcock et al. (2021) in a comprehensive overview of key rotor parameters, deploying bottom-fixed turbines in depths greater than 50 m with the aim of positioning the hub height in the upper half of the water column would require tall and thick support structures. The increasing size of support structures leads to an increase in thrust applied to the flow, which detrimentally impacts the resource due to the decrease in flow velocity, and also becomes uneconomical. Therefore, the option of a floating foundation is considered because the inversion of the support structure allows for its length to be reduced, along with the diameter of the support structure, whilst still remaining in the upper portion of the water column. Consequently, all turbines in any array deployed in regions with an average depth greater than 50 m adhere to support structure dimensions pertaining to floating foundations; otherwise, support structures are sized in accordance with requirements for bottom-fixed turbines.

### Model inputs

The turbine density for each array area was determined by calculating the number of turbines based on spacing that achieves approximately 0.16 blockage. A blade optimised for 0.16 blockage, following the design of Cao et al. (2018), was selected as the most suitable design for this study. The coefficients of lift and drag of the Risø-A1-24 aerofoil were obtained from Chen et al. (2019b), adapted from Wimshurst and Willden (2016). The thrust applied by the cylindrical, bottom-fixed, support structure is calculated as $F_S = 0.5 \rho C_{D,S} A_S u^2$, with drag coefficient $C_{D,S}=1.2$ (Muchala and Willden 2017). The frontal area of the support structure, $A_S$, is the product of the support structure diameter $D_S$ and the length of the support structure $L_S$. No explicit modelling is carried out for wake effects beyond those which are captured in the shallow water model.

In this study, turbines in arrays located in regions with an average depth exceeding 50 m are designed with support structures suitable for floating foundations; in shallower regions, support structures are sized to meet the specifications for bottom-fixed turbines. In both cases, the drag generated by the vertical tower of the support structure is included. For bottom-fixed turbines, the total length of the support structure is calculated as the sum of the turbine radius and the clearance from the seabed to the blade tip. Minimum clearances between the rotor tip and both the seabed and sea surface for bottom-fixed turbines are determined based on the guidelines provided by Meygen (2012): 5 m blade tip to seabed and 8 m blade tip to sea surface. For floating turbines, guidance for the rotor tip to sea surface clearance is taken from the Orbital O2 device, where the nacelle is positioned 14 m below sea surface for a 20 m diameter turbine, leaving approximately 4 m clearance between the blade-tip and sea surface (Orbital Marine 2022; European Marine Energy Centre 2023).

The diameter of the support structure is based on a 0.2 ratio between $A_S$ and the rotor area, $A_T$, following Muchala and Willden (2017) for a 20 m diameter turbine. When turbines are deployed in areas with greater depth than the minimum clearance requirements, the diameter of the support structure is increased according to the increased length of the support structure, as described in Patel et al. (2024).

## Results and discussion

Four arrays are sequentially defined across the Pentland Firth, in addition to array 1 in the Inner Sound. The specification of turbines across the five arrays, defined through the extended heterogeneous framework, is presented in Fig. 3. The turbines in array 1 are assumed to be bottom-fixed because the average bathymetry across the array is less than 50 m and the turbines in arrays 2–5 are assumed to be floating. The support structure sizes for each turbine reflect these assumptions. The turbines that make up array 2, array 4 and array 5, are all homogeneous in diameter (20 m) because the areas have bathymetry deep enough to accommodate this size of turbine rotor and meet minimum clearance requirements. Array 3 primarily consists of 20 m diameter turbines, although some 15 m diameter turbines are deployed in the north-eastern part of the array.

Five cases are defined, extending from the case discussed in Patel et al. (2024). Each case is established with the addition of an array, following the sequential order of identification outlined in Sect. 5.1. With the deployment of each new array the number of turbines and total swept area increases in each case.

### Defining arrays

**Fig. 4:**
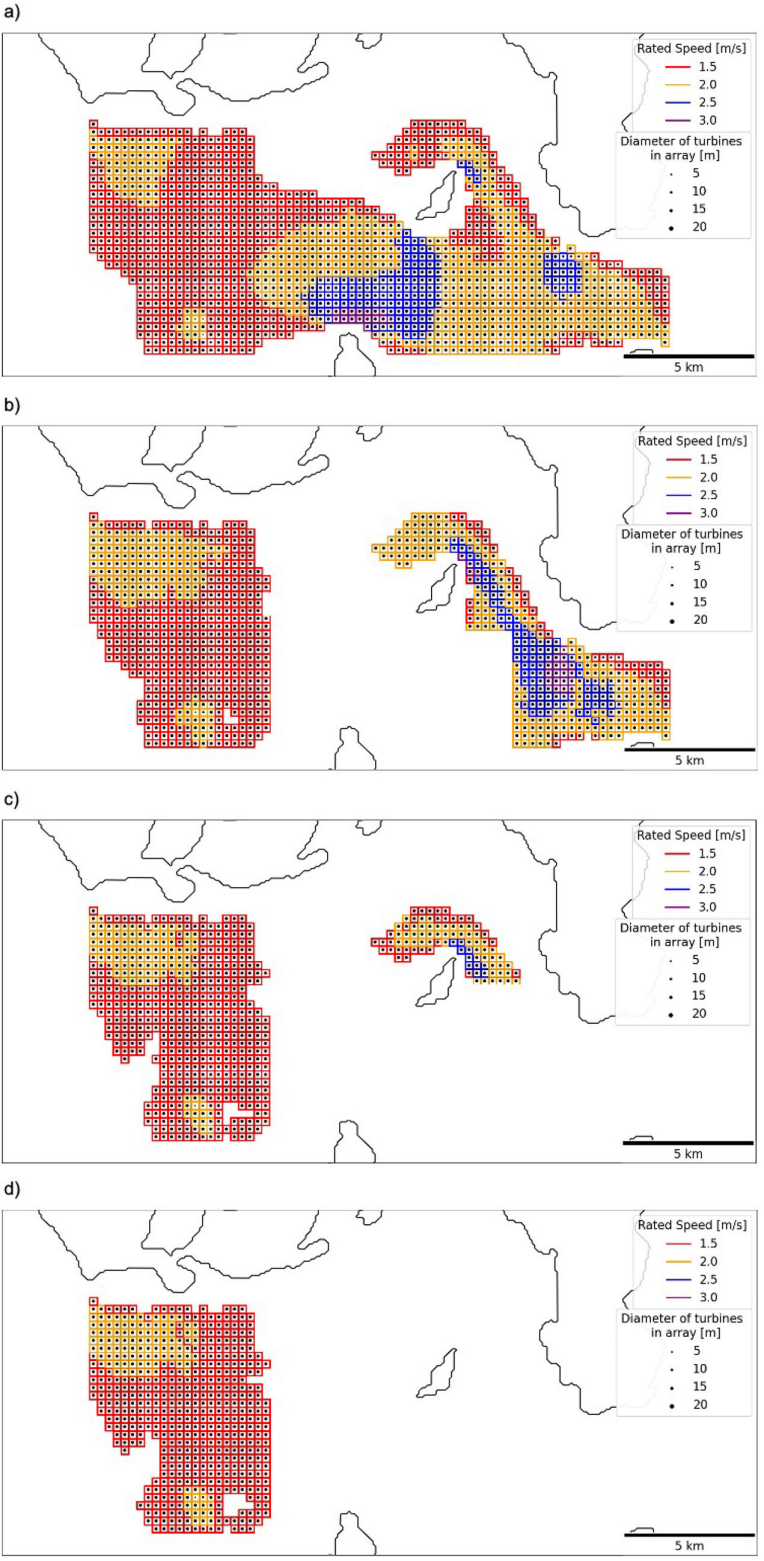
Specification of turbines within potential remaining array areas to define and deploy (**a**) array 2, (**b**) array 3, (**c**) array 4 and d) array 5 as each additional array is deployed. The areas occupied by deployed arrays, and buffer zone, are omitted from potential development areas

Figure 4 presents the available areas for development as arrays are deployed and the change of turbine specifications with the addition of each array is visible. Array 2 is situated north of the Inner Sound, between Stroma and Swona, selected on the basis that it is the only area with turbines operating at a rated speed of 3 m/s (Fig. 4a), which indicates a high flow velocity and concentration of the resource.

When the heterogeneous framework is applied, based on the updated flow field, the change in turbine specifications across the available areas for development as the resource responds to the deployment of arrays 1 and 2 can be seen in Fig. 4b, in comparison to Fig. 4a. Array 3 is defined around the area with turbines operating with rated speed of 3 m/s, located east of array 2 (Fig. 3).

Following the implementation of the three arrays, there are no more areas with turbines specified to operate at rated speed of 3 m/s when the heterogeneous framework is re-applied (Fig. 4c). From the remaining available areas for development, array 4 is defined around the island Swona, with turbines operating at a maximum rated speed of 2.5 m/s. Subsequently, array 5 is defined on the north-west side of the site, in an area where the maximum rated speed turbines are operating at 2 m/s.

**Table 1:** Overview of the size of arrays in each case and the average power, capacity factor, power per swept area and power per turbine for each array

		Array 1	Array 2	Array 3	Array 4	Array 5	Overall
	Number of array areas	81	309	231	92	124	837
	Swept area [m$^2$]	136,945	518,397	388,002	154,514	208,257	1,406,115
	Number of turbines	2134	1652	1260	492	663	6201
	Installed capacity [MW]	260	1670	830	320	400	3490
1 Array	Capacity factor	0.31	–	–	–	–	0.31
	Average power [MW]	75.32	–	–	–	–	75.32
	Power per swept area [kW/m$^2$]	0.55	–	–	–	-	0.55
	Power per turbine [kW/turbine]	35.30	–	–	–	–	35.3
2 Arrays	Capacity factor	0.38	0.47	–	–	–	0.45
	Average power [MW]	91.33	767.07	–	–	–	858.40
	Power per swept area [kW/m$^2$]	0.67	1.48	–	–	-	1.31
	Power per turbine [kW/turbine]	42.80	464.33	–	–	-	228.5
3 Arrays	Capacity factor	0.36	0.45	0.48	–	–	0.45
	Average power [MW]	89.89	739.02	347.56	–	–	1176.47
	Power per swept area [kW/m$^2$]	0.66	1.43	0.90	–	–	1.13
	Power per turbine [kW/turbine]	42.12	447.35	275.84	–	–	235.6
4 Arrays	Capacity factor	0.37	0.45	0.47	0.39	–	0.44
	Average power [MW]	90.53	737.38	336.95	120.55	–	1285.41
	Power per swept area [kW/m$^2$]	0.66	1.42	0.87	0.78	–	1.07
	Power per turbine [kW/turbine]	42.42	446.35	267.42	245.01	–	234.7
5 Arrays	Capacity factor	0.36	0.46	0.46	0.37	0.40	0.43
	Average power [MW]	90.15	741.89	330.25	113.97	154.28	1430.54
	Power per swept area [kW/m$^2$]	0.66	1.43	0.85	0.74	0.74	1.02
	Power per turbine [kW/turbine]	42.24	449.09	262.11	231.64	232.69	233.4

### Incremental development of the Pentland Firth

The average power and capacity factor of each array is presented in Table 1 for each case. The average power refers to the time-averaged power over the spring-neap cycle. Considering the varying turbine diameter and number of turbines across the arrays, assessing the average power per turbine and per swept area provides deeper insights into array performance and are presented for each array in each case in Table 1. The table also provides details on the number of array areas, swept area, number of turbines and installed capacity both individually and collectively.

**Fig. 5:**
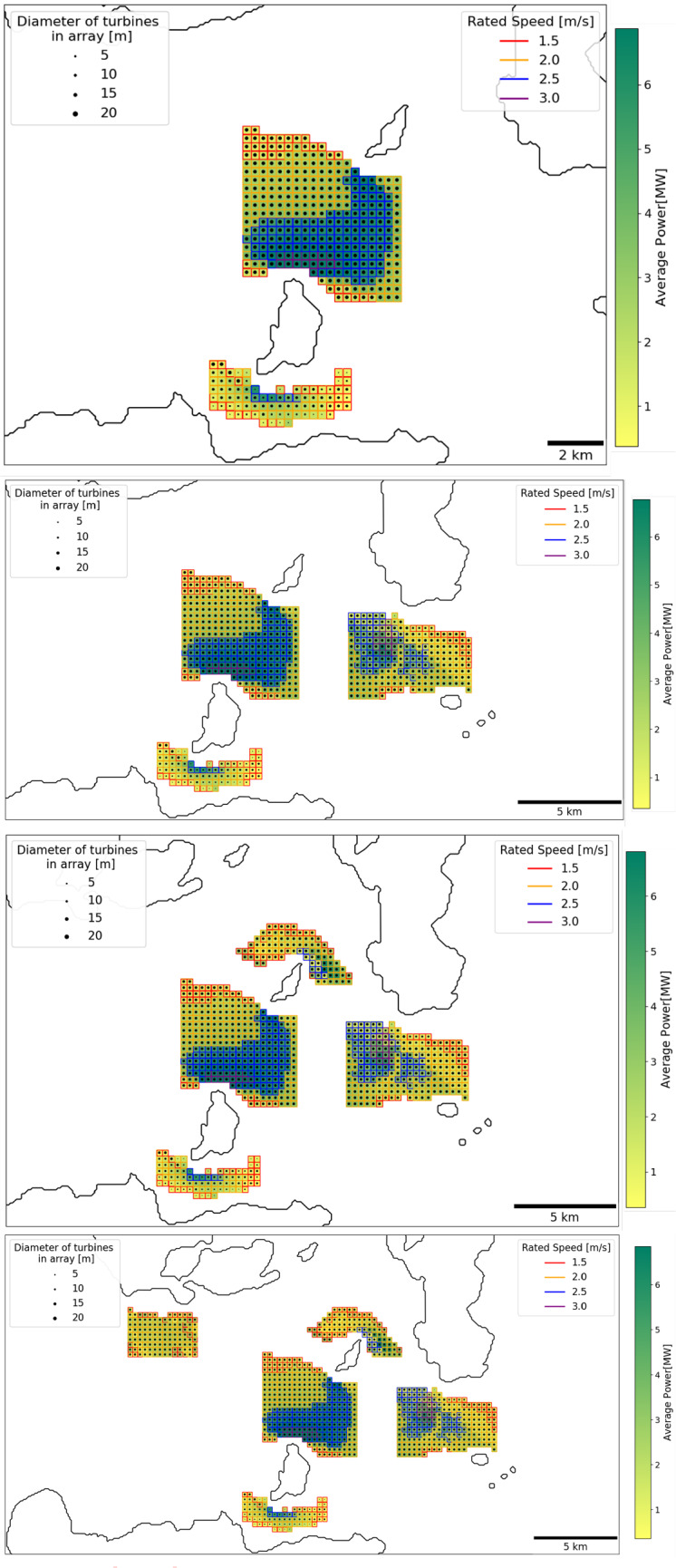
Average power [MW] generated by the arrays over the spring-neap cycle for each deployment scenario. The rated speed of turbines in each array area is illustrated by the edge colour of the square and the diameter of the turbines is indicated by the size of the black circle

**Fig. 6:**
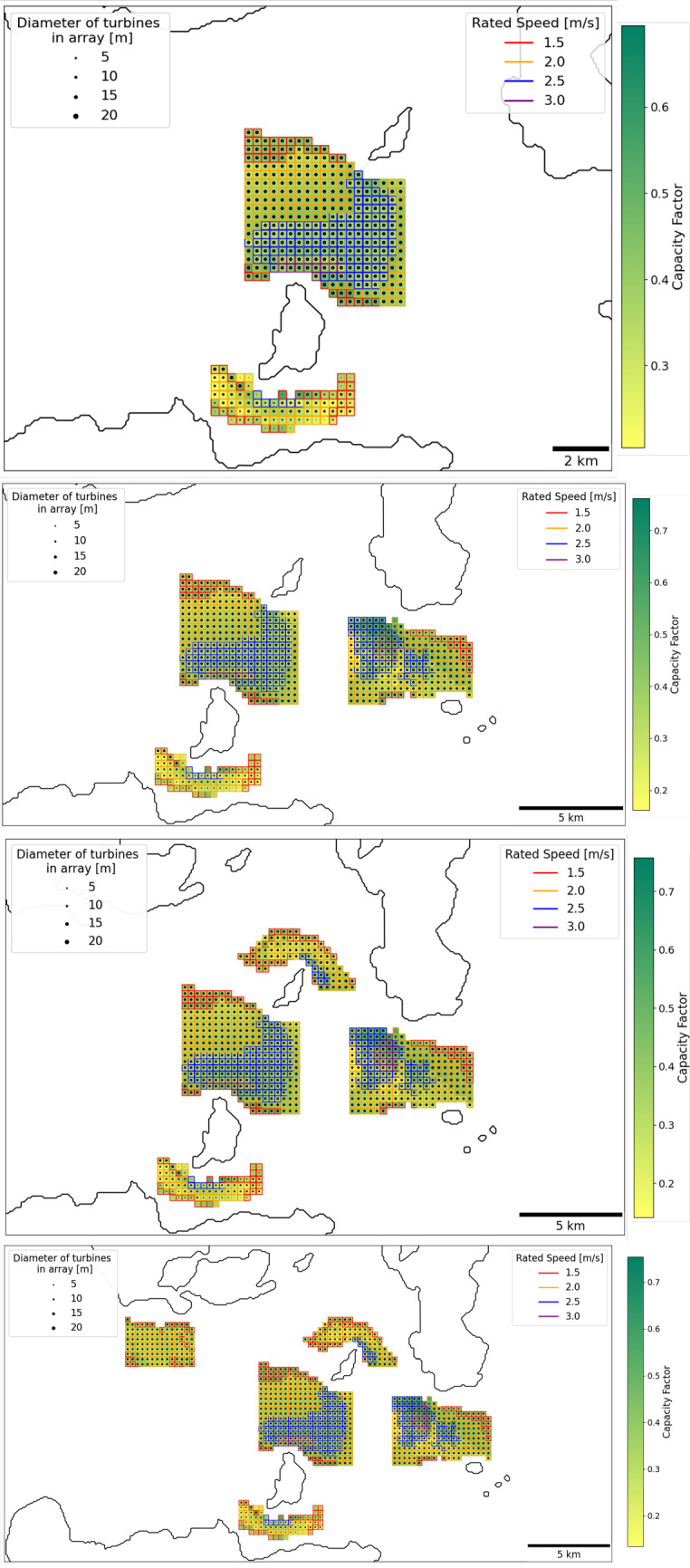
Capacity factor of the arrays for each deployment scenario. The rated speed of turbines in each array area is illustrated by the edge colour of the square and the diameter of the turbines is indicated by the size of the black circle

The average power and capacity factor for each case are also presented in Figs. 5 and 6 to visualise the distribution of the metrics spatially across the site and in context of the turbine specification in each case.

Deploying turbines beyond the Inner Sound (array 2) results in a 15% increase in power for array 1, averaged over the spring-neap cycle. The addition of array 3 results in a minor decrease in the power yielded by arrays 1 and 2 (2 and 4% respectively). When array 4 and array 5 are deployed, there is a minimal increase in power for array 1 in comparison to the 3 array case. The average power yield for array 1 varies by less than 1% between the case with 3 arrays and 5 arrays. There is a 5% decrease in power yielded by array 3 with the addition of array 4 and 5. Array 4 also experiences a 5% decrease in power due to the presence of array 5.

The average capacity factor of the arrays ranges from 31 to 45%, depending on the number of independent arrays deployed. Array 2 exhibits the highest power per swept area and power per turbine, along with the largest installed capacity amongst the arrays. Array 1 demonstrates the smallest power per turbine, power per swept area, and installed capacity.

**Fig. 7:**
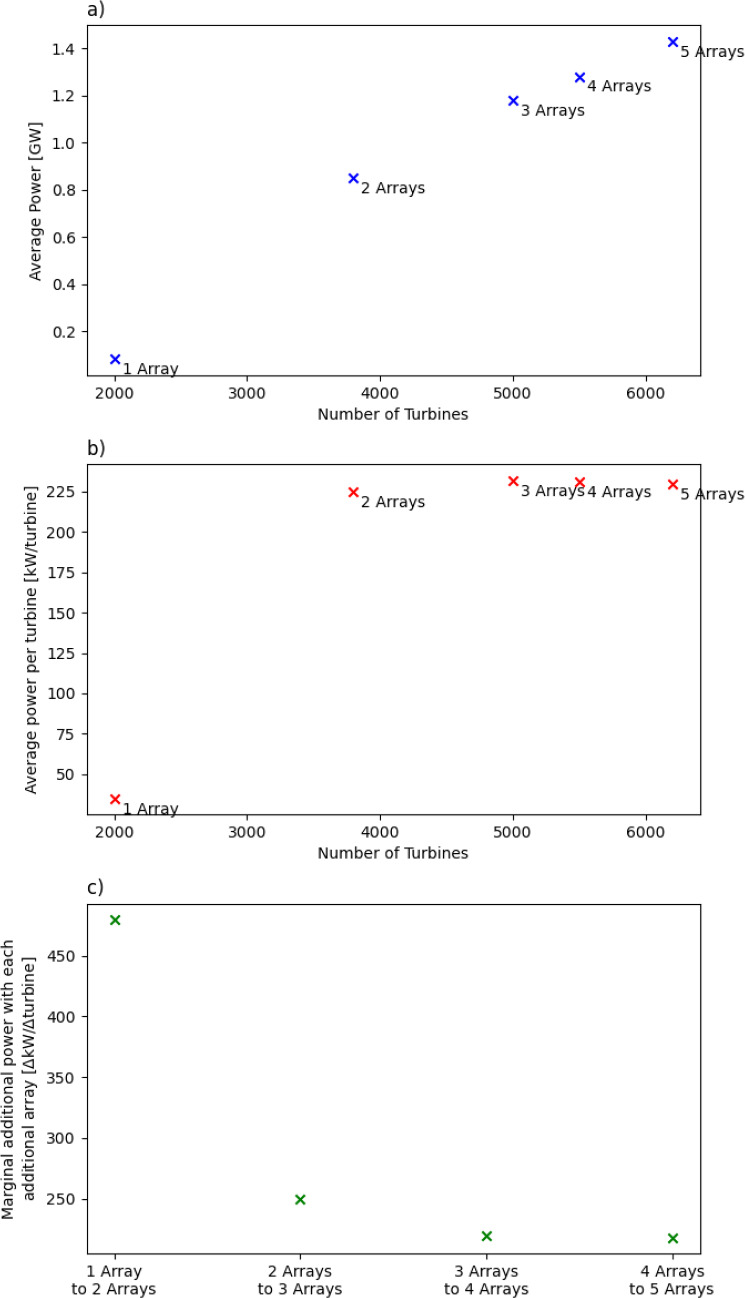
Relationship between power output and turbine deployment across incremental development cases. (**a**) Total average power versus the number of turbines for each deployment case; (**b**) Average power per turbine versus the number of turbines for each deployment case; (**c**) Marginal additional power gained with the inclusion of each new array. These plots illustrate how overall and per-turbine performance evolves as arrays are added sequentially across the Pentland Firth

Figure 7a presents the total average power yielded by the arrays in each case against the total number of turbines deployed. Figure 7b illustrates the power per turbine for increasing number of turbines in each case. To assess the relative differences between each point on the curve in Fig. 7a, the change in power divided by the change in the number of turbines between cases, essentially the gradient of the line between points on Fig. 7a, is depicted in Fig. 7c. The ratio of instantaneous power and instantaneous thrust (i.e., power per thrust) is proportional to the overall power for each case, therefore, the average power per thrust per turbine demonstrates the same trends as power per turbine.

As more turbines are deployed, through the inclusion of an additional array in each case, the total power output increases, evidenced in Fig. 7a. As demonstrated in Fig. 7b, the power per turbine increases to 230 kW per turbine, from 35 kW per turbine, with the addition of array 2, and remains almost constant as arrays are added until there are 5 arrays. The low power per turbine for array 1 is due to the smaller diameter turbines in comparison to the other arrays. When the change in power over the change in turbine is plotted (Fig. 7c) between each case, it becomes evident that the gradient of the average power versus the number of turbines plot decreases at a progressively slower rate and becomes almost constant. This implies that power increases at the same rate, as more turbines are deployed in arrays across the site.

### Array interactions

**Fig. 8:**
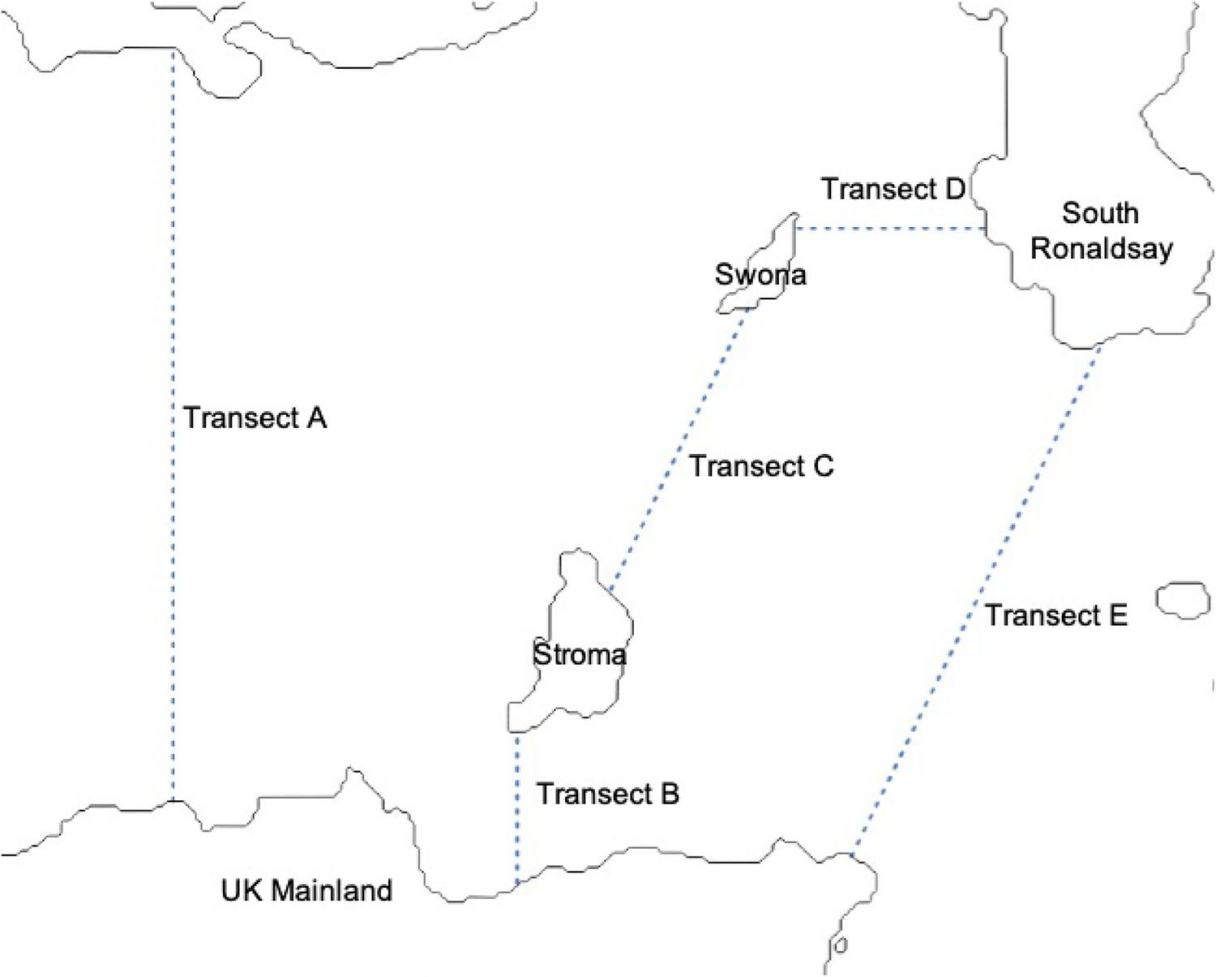
Labelled transects (A–D) across the Pentland Firth used to evaluate changes in flow rate amplitude. Each transect spans a key channel or sub-channel where tidal flow is expected to be influenced by turbine deployment. These transects provide reference lines for assessing hydrodynamic impacts under different array development scenarios

To evaluate the influence of energy extraction through turbines on flow dynamics and subsequent effects on the average power produced by each array, the flow rate across five transects illustrated in Fig. 8 are considered. Table 2 presents the percentage change in flow rate amplitude for each case with respect to the ambient case across each transect. The relative changes in flow rate amplitude between cases is one way to demonstrate the impact of developing arrays in the Pentland Firth on the flow across the site.

When array 1 is deployed, a 9.8% decrease in maximum flow rate is observed across transect B, which is extended across the Inner Sound and in the same location as array 1. The flow rate amplitude across transect C increases by 0.7%. transect C, positioned between Stroma and Swona, is located in the area identified to develop array 2 due to concentration of the resource north of Stroma, indicated through the rated speed from the heterogeneous design framework (Fig. 5.1a).

**Table 2:**
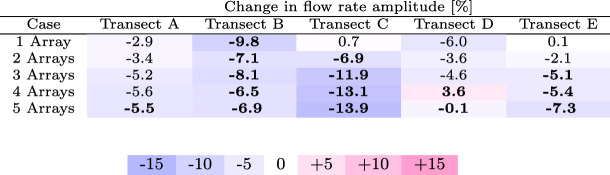
Percentage change in flow rate amplitude with respect to the ambient case

Values in bold are where the deployed arrays coincide with the transect in each case

The extraction of energy through arrays 1 and 2 results in a 3% increase in flow rate amplitude across transect B, contributing to the 15% increase in power yielded by array 1 compared to the case with 1 array only. This illustrates the positive interaction between array 1 and array 2, which are situated in parallel sub-channels of flow, similarly observed in studies by Draper et al. (2014b, 2014a); Coles et al. (2017). An 8% decrease in flow rate amplitude is observed across transect C between the 1 array and 2 arrays cases and attributed to the presence of array 2. Array 2 has a significantly larger installed capacity of 1670 MW, which explains the significant decrease in flow rate due to the resistance imposed on the flow.

Comparing Fig. 4a and b, it is evident that the deployment of array 2 in combination with array 1, results in an increased rated speed of turbines in the remaining areas for development on the east-side of the site. The increased resistance between Swona and Stroma leads to an increase in flow velocity around the north and east of Swona. Considering the change in flow rate amplitude across transect D, which is located east of Swona, there is a 3% relative increase in flow rate amplitude between the 1 array and 2 arrays cases. This demonstrates the deployment of 2 arrays also leads to a positive interaction across transect D.

Conversely, Transects E and A, located in series with array 2, experience a decrease in flow rate amplitude across both transects following the deployment of array 2. Despite the apparent relative decrease in flow rate amplitude across transect E, array 3 is defined in the north part of the transect where the rated speed of turbines increases due to the presence of array 2. The area east of array 2 appears to experience increased flow velocity due to the acceleration around Swona. However, the area east of array 1 and south east of array 2 experiences a decrease in flow velocity due to the combined negative interference of arrays 1 and 2. The Ness of Duncansby lease lies in this area but the study suggests this is a relatively low resource area.

**Fig. 9:**
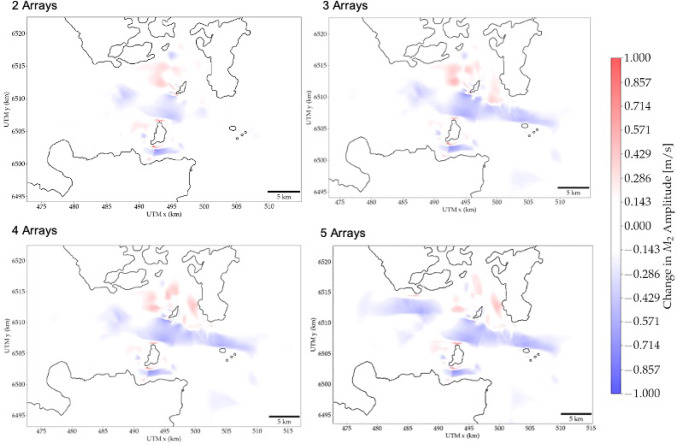
Change in amplitude of the M$_2$ semi-diurnal lunar tidal constituent for each development case relative to the ambient (no-turbine) scenario. Red demonstrates an increase in change in amplitude and blue represents a decrease

Array 4 is defined around the north and east side of Swona. When three arrays are deployed, the flow rate in this region decreases slightly, evidenced by a 1% decrease in flow rate across transect D and lower rated speed specification of turbines across the site between Fig. 4b and c. The flow rate amplitude across transect D increases with respect to the ambient case. It is expected that the presence of an array in the area where the transect is located would relate to a decrease in flow rate relative to the ambient flow, therefore the results seems counter-intuitive. Figure 9 presents the change in amplitude for the M$_2$ constituent in each case relative to the ambient case. The plot reveals an increase in the M$_2$ velocity amplitude on the east side of transect D, which is not occupied by the array. This indicates the increase in flow rate across transect D is due to the bypassing flow velocity increasing. Across other transects, the increase and decrease in flow rate amplitude relative to the 3 arrays case reflects in the power yielded from the relative array coinciding over each transect.

The final array, array 5, is positioned in the north-west of the site and comprises turbines with rated speeds of 1.5 m/s and 2 m/s. When all five arrays are deployed, the flow rate amplitude decreases by 13.9% across transect C relative to the ambient case, marking the most significant decrease across all cases and transects. This reduction is primarily attributed to the presence of array 2, which is situated in the same area as transect C and is in series with two other arrays, array 3 and array 5. Consequently, the reduction in flow velocity through outer sound is heightened, due to negative interference, as illustrated in Fig. 9.

### Pentland Firth development

The cases presented in this study propose a gradual expansion of tidal stream energy developments across the Pentland Firth, which prioritises the need to harness tidal energy as a complementary renewable energy source to achieve net zero targets (Climate Change Committee 2020). It is unlikely that the site will be developed all at once, and the method for identifying each array in this study demonstrates how the site can be incrementally developed as lease sites and seabed priorities evolve. Therefore, as every new array is defined sequentially, cases were run with the addition of each array. This allows the opportunity to consider how the resource responds to additional developments and the power yielded by the combination of arrays in each case if the site is developed in the same successive order, which is presented in Table 1.

Previous studies have examined the dynamics of multiple arrays across a site and found significant positive and negative interactions between arrays, dependent on whether they are arranged in parallel or series. However, in the arrays modelled in this study, whilst there are observable interactions between arrays in terms of power and flow rate amplitude across different areas of the site, their overall impact is not as significant. One notable exception is the effect on array 1 when array 2 is additionally deployed, which experiences a significant positive interaction. This is partly due to the installed capacity of the other 4 arrays being more than half of array 2, therefore imposing less resistance on the flow. Conversely, arrays 2–4 only experience up to 6% fluctuation in power output between cases (array 5 is not comparable because it is the last array to be deployed).

Table 3 details the number of arrays that are in parallel and series for each case. Array 2 is a critical array for the interactions between arrays because all positive and negative interference is due to the interaction with array 2, which is centrally located in the site. In the 5 arrays case, array 2 is in series and parallel with 2 arrays respectively, therefore it is both experiencing and causing these effects. The significantly increased installed capacity of array 2, in comparison to the other arrays, causes the interference to be enhanced.

It has also been demonstrated in previous studies that increasing the number of turbines at a site, and therefore increasing the thrust imposed on the flow, leads to diminishing returns in power after a certain level of deployment (Draper et al. 2014b; Funke et al. 2016; O’Hara Murray and Gallego 2017). Vennell et al. (2015) emphasises that the energy available to an array is limited and therefore, the power per turbine will decrease with increasing numbers of turbines after a certain point. However, as demonstrated in Fig. 7a, in this study the power does not demonstrate diminishing returns when the overall resistance is increased through the addition of more arrays and turbines. This implies that for this site, a realistic deployment will not be large enough to generate the diminishing return seen in more idealised models. A steep increase in power per turbine from the case with 1 array to the case with 2 arrays is apparent from Fig. 7b. This occurs because array 1 is made up of smaller diameter turbines. Therefore, to achieve the same capacity, more turbines are required compared to arrays 2, 3, 4, and 5, which are made up primarily of 20 m turbines. The power per turbines remains almost constant after this point, rather than decreasing as in other studies, which highlights the benefit of identifying the arrays sequentially, based on the flow field of the previous deployment. As a result, the array specification adapts to how the resource is changing. Therefore, when multiple arrays are deployed, the rate of power per turbine remains constant up to the 5 arrays being deployed (Fig. 7b). The sequential identification of arrays also contributes to the positive and negative interactions between arrays being less significant, because the resistance of the turbines in each array and the effect on the flow is accounted for in the definition of the next array, which minimises the effect without eliminating it. The change in power may diminish with a greater turbine density across the arrays. Therefore, a set of cases are considered to investigate the impact of increasing in turbine density for all five arrays, with the aim of testing the upper limit of power before diminishing returns are observed (Sect. 5.6).

**Table 3:** Combination of arrays in parallel and series for each case

	Number of arrays	
	Parallel	Series
1 Array	–	–
2 Arrays	2	0
3 Arrays	2	1
4 Arrays	3	1
5 Arrays	3	3

**Fig. 10:**
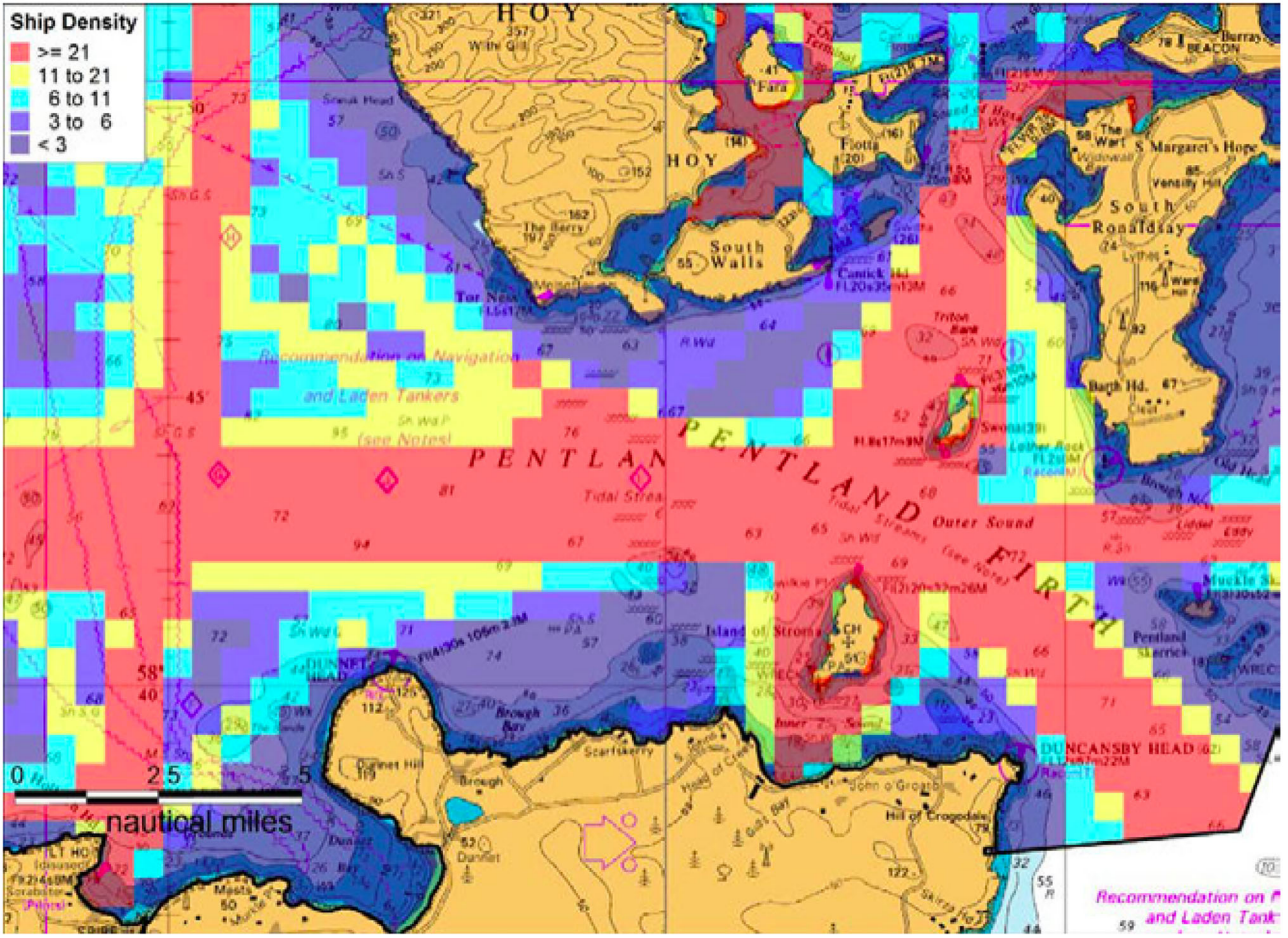
Ship traffic density across the Pentland Firth based on AIS (automatic identification system) tracking data. Source: Marine Scotland (2012)

### Seabed usage constraints

A key factor for developing commercial tidal stream arrays is ensuring navigational channels and marine life passages are unobstructed, forming part of the decision for defining lease sites. The Scottish Government proposed ‘Highly Protected Marine Areas’ (HPMAs) to cover 10% of Scotland’s marine areas by 2026 (Marine Scotland Science 2022). The HPMAs were proposed to enable recovery of marine ecosystems and encourage a thriving environment. Under the proposal, any ‘exploratory activity or construction of new infrastructure’ for renewable energy would not be allowed in HPMAs. However, all shipping, including shipping associated with the development of renewable energy projects would be allowed. The proposal noted the need for offshore renewable energy and therefore, suggested that any existing agreements for renewable energy projects would be excluded when defining the HPMAs and remain unaffected. The Scottish Government conducted a consultation on the proposal, from December 2022 to April 2023, and received strongly divided responses. Therefore, as of November 2023, they are not implementing the policy for HPMAs (Scottish Government 2023).

Despite HPMAs not being implemented, a significant seabed usage constraint for tidal stream energy developments that remains is the need to ensure navigation routes through the Pentland Firth. To inform the marine spatial planning pilot of the Pentland Firth and Orkney Waters (Marine Scotland 2016), the Scottish Government conducted a shipping study to ensure future developments in the area do not impede on critical existing activities (Marine Scotland 2012). The density of ships across the Pentland Firth is presented in Fig. 10 (Marine Scotland 2012).

**Fig. 11:**
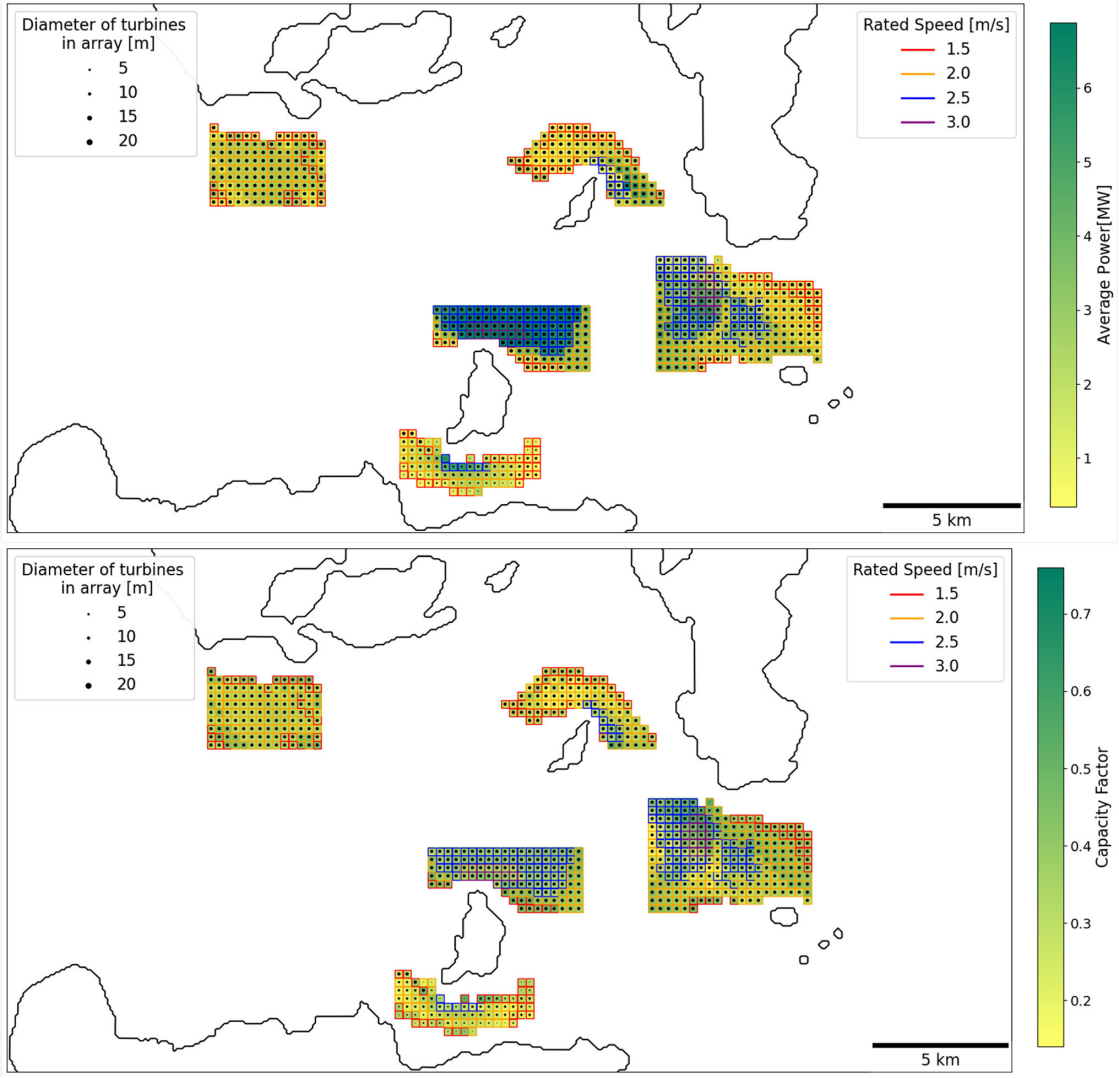
Average power and capacity factor of all arrays in the shipping lane accommodation case. The rated speed of turbines in each array area is illustrated by the edge colour of the square and the diameter of the turbines is indicated by the size of the black circle

While Fig. 10 depicts multiple areas with high shipping density, the Outer Sound is a critical area for commercial ships (e.g. container ships) navigating north of the Scottish mainland. In the cases presented in this study, array 2 occupies the majority of the Outer Sound (Fig. 3), and leaves no navigation route. Therefore, a more practical case is presented where array 2 is limited to accommodate a large shipping lane through the Outer Sound. This case allows 5 arrays to be developed across the Pentland Firth without significant rerouting of ships, which the Chamber of Shipping emphasised as a key concern in a shipping and navigation consultation undertaken by Meygen (2012) regarding the Inner Sound lease site development. The navigation route is approximately 2.8 km in width, which is reasonable for the size of ship that passes through and the strong currents in the area (Meygen 2012; Marine Scotland 2012). Jones et al. (2022) reported that 36% of the world’s shipping carries energy products, which are predominantly fossil fuels. An energy transition to renewable sources will see the shipping of energy products fall, and could allow for the re-prioritisation of the seabed and reduce the number of large shipping lanes required. Figure 11 presents the update to array 2 and the average power and capacity factor of the five arrays for the shipping channel accommodation case.

Table 4 presents the average power, capacity factor and size of the arrays in terms of number of array areas, swept area, number of turbines and installed capacity for the 5 arrays case and the case adapted for shipping. In comparison to the five arrays case, the shipping case features only 38% of the number of array areas, turbines, and swept area in array 2. Despite this reduction, the installed capacity of array 2 in the shipping case is 47% of array 2 in the five arrays case. The areas of array 2 that are retained in the shipping case are characterised by higher rated speeds and greater resource, compared to the north part of the Outer Sound. This strategic placement allows for the removal of more than half of array 2 to accommodate ships in areas with lower flow velocity, whilst retaining turbines in regions with higher flow velocity that produce 50% of the power than an array more than double its size. As a result, the power per swept area and power per turbine for array 2 increases in the shipping case.

**Table 4:** Results for the five arrays case and the shipping case

		Array 1	Array 2	Array 3	Array 4	Array 5	Overall
5 arrays	Number of array areas	81	309	231	92	124	837
	Swept area [m$^2$]	136,945	518,397	388,002	154,514	208,257	1,406,115
	Number of turbines	2134	1652	1260	492	663	6201
	Installed capacity [MW]	260	1670	830	320	400	3490
	Capacity factor	0.36	0.46	0.46	0.37	0.40	0.43
	Average power [MW]	90.15	741.89	330.25	113.97	154.28	1430.54
	Power per swept area [MW/m$^2$]	0.66	1.43	0.85	0.74	0.74	1.02
	Power per turbine [MW/turbine]	42.24	449.09	262.11	231.64	232.69	233.4
Shipping	Number of areas	81	117	231	92	124	645
	Swept area [m$^2$]	136,945	195,934	388,002	154,514	208,257	1,083,652
	Number of turbines	2134	625	1260	492	663	5174
	Installed capacity [MW]	260	780	830	320	400	2600
	Capacity factor	0.36	0.49	0.48	0.34	0.42	0.44
	Average power [MW]	87.89	371.49	344.25	103.82	163.79	1071.25
	Power per swept area [kW/m$^2$]	0.64	1.90	0.89	0.67	0.79	0.99
	Power per turbine [kW/turbine]	41.19	594.39	273.21	211.02	247.05	207.0

**Fig. 12:**
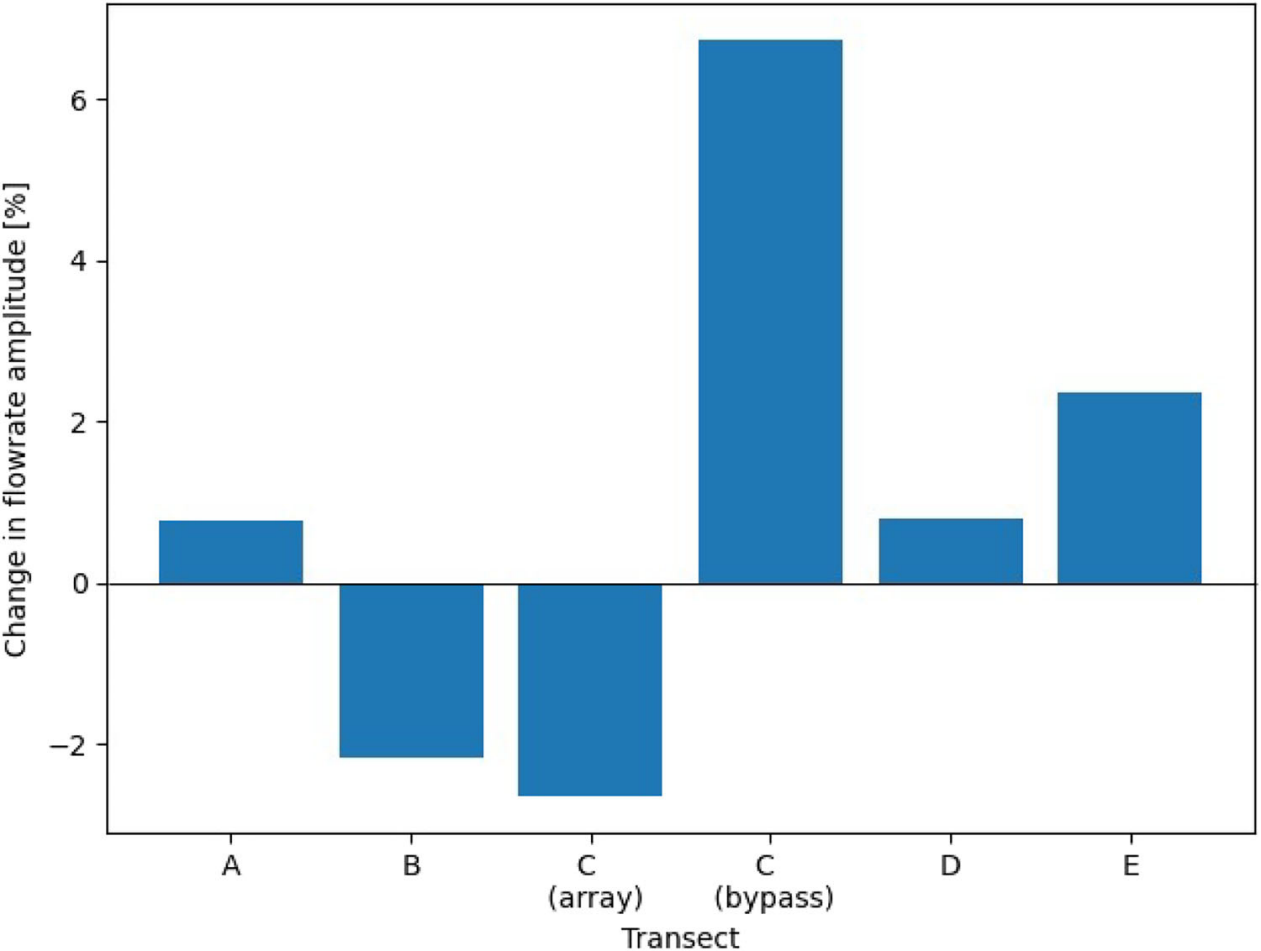
Percentage change in flow rate amplitude across five transects for the shipping case with respect to the five arrays case. Transect C is presented in two portions, the portion occupied by array 2 in the shipping case and the portion bypassing the array

The adjustment to array 2 for the shipping case impacts the interaction between arrays and the power produced by each, which is reflected in the change in flow rate amplitude across the five transects between the five arrays case and the shipping case. Figure 12 presents the change in flow rate amplitude for the shipping case with respect to the five arrays case. The flow rate across transect C is evaluated separately over the portion that is occupied by array 2 in the shipping case and the remaining proportion of the transect.

The adjustment made to array 2 for the shipping case results in increased power for arrays 3 and 5 by up to 4%, which are located across Transects E and A respectively. The flow rate amplitude across both transects also increases (up to 2.4%). In the five arrays case, arrays 3 and 5 are in series with array 2, however, in the shipping case, they are no longer in direct obstruction of each other, which is why there is an increase in power for each of the arrays in the shipping case relative to the original case.

The power yielded by array 1 and array 4 decreases in the shipping case, with respect to the five arrays case. The flow rate amplitude across transect B, where array 1 is located, decreases by 2.16% and the power produced by array 1 also decreases by 3% in the shipping case. However, despite a small increase (less than 1%) in flow rate amplitude across transect D, the power from array 4, located over the transect, decreases by 9%. This is attributed to the fact that array 4 partially coincides with transect D, where the flow rate decreases, but the flow bypassing array 4 experiences an increase in velocity, and this bypassing flow rate is enhanced when array 2 is adjusted for the shipping case.

**Fig. 13:**
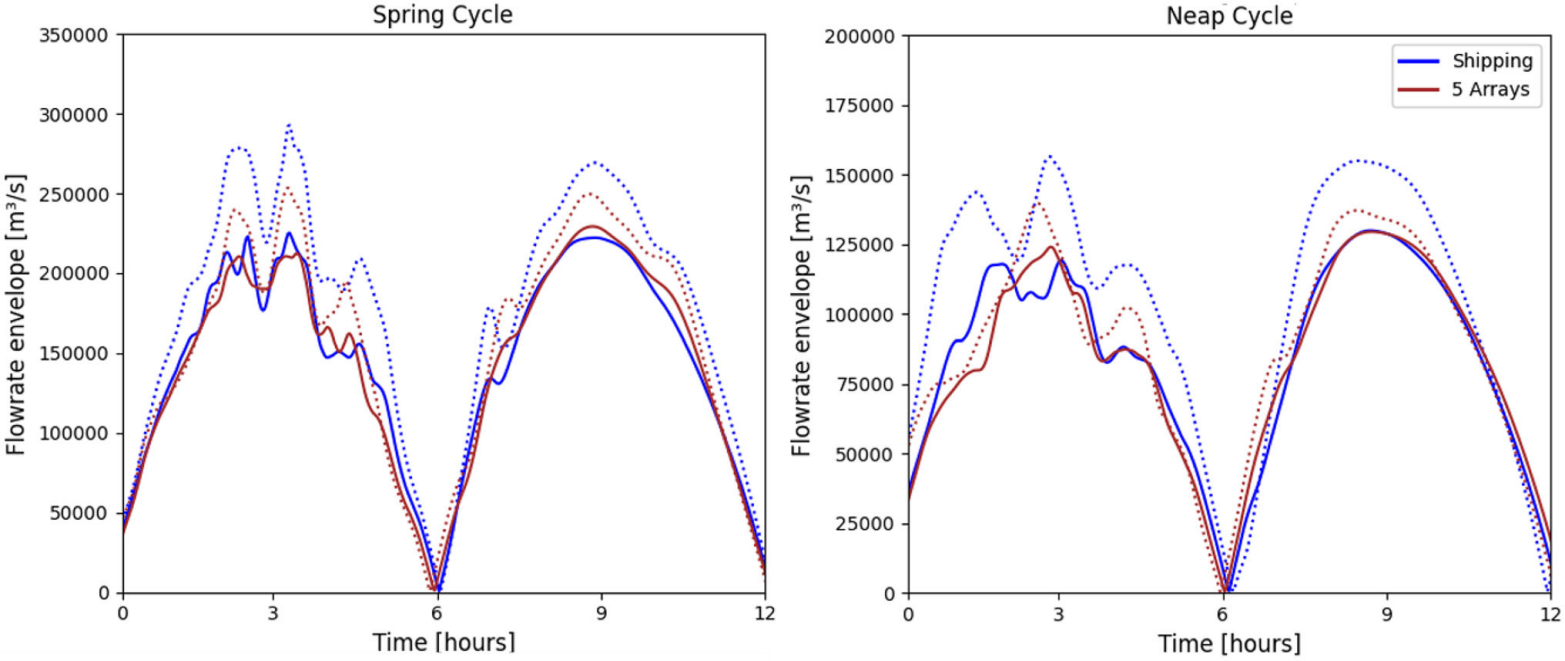
Envelope of the flow rate across transect C for the shipping case and five arrays case, solid line representing the proportion of the transect occupied by array 2 in the shipping case and the dotted line representing the proportion of transect on the north side of array 2 during a spring cycle and neap cycle

A similar effect is observed across transect C for the shipping case because a portion of the transect is occupied by array 2 and the other portion experiences the flow bypassing array 2 with an increased velocity. This is demonstrated in Fig. 12, where it can be seen that the flow rate amplitude across the bypass portion of transect C increases by 6.72% in the shipping case compared to the 5 arrays case and decreases by 2.64% in the array portion of transect C. The envelope of the flow rate across the array and bypass portion of transect C is plotted in Fig. 13. In the shipping case, the flow rate amplitude across the bypass portion is 19% greater than the flow rate amplitude through the array. Compared to the five arrays case, where the ‘bypass’ portion is occupied by array 2 and only experiences an 8% increase compared to the ‘array’ portion.

As discussed previously, the increase in flow rate bypassing array 2 in the shipping case causes the negative interference for arrays 3 and 5, to be less significant in comparison to the five arrays case. Similarly, the positive interference for arrays 1 and 4, due to array 2, are also diminished. This is because the flow in the parallel channels that the arrays are located in, do not experience as great an increase in flow velocity because the width of the Outer Sound is much less significantly blocked by array 2 in the shipping case. Therefore, the flow rate through the bypass portion of transect C increases. As discussed in Sect. 5.4, the interference between arrays is mostly dependent on array 2 due to its positioning and significant installed capacity in the non-shipping cases. Reducing the capacity and spatial extent of array 2 in the shipping case, demonstrates that it was the driving force for these interactions because they are diminished as the array is diminished (both in terms of installed capacity and occupation of the Outer Sound).

### Turbine density

To consider an alternative scale of development, the impact of increasing turbine density in all the arrays defined across the Pentland Firth is examined. This analysis aims to explore the extent of development that could take place across the Pentland Firth and investigate an upper limit of exploitation, based on the inclusion of practical aspects considered in the present paper. Increasing the turbine density means the thrust imposed on the flow due to turbines also increases, therefore, the power will only increase up to a certain point until too many turbines are deployed (Funke et al. 2016), which allows the upper limit of development to be investigated.

**Fig. 14:**
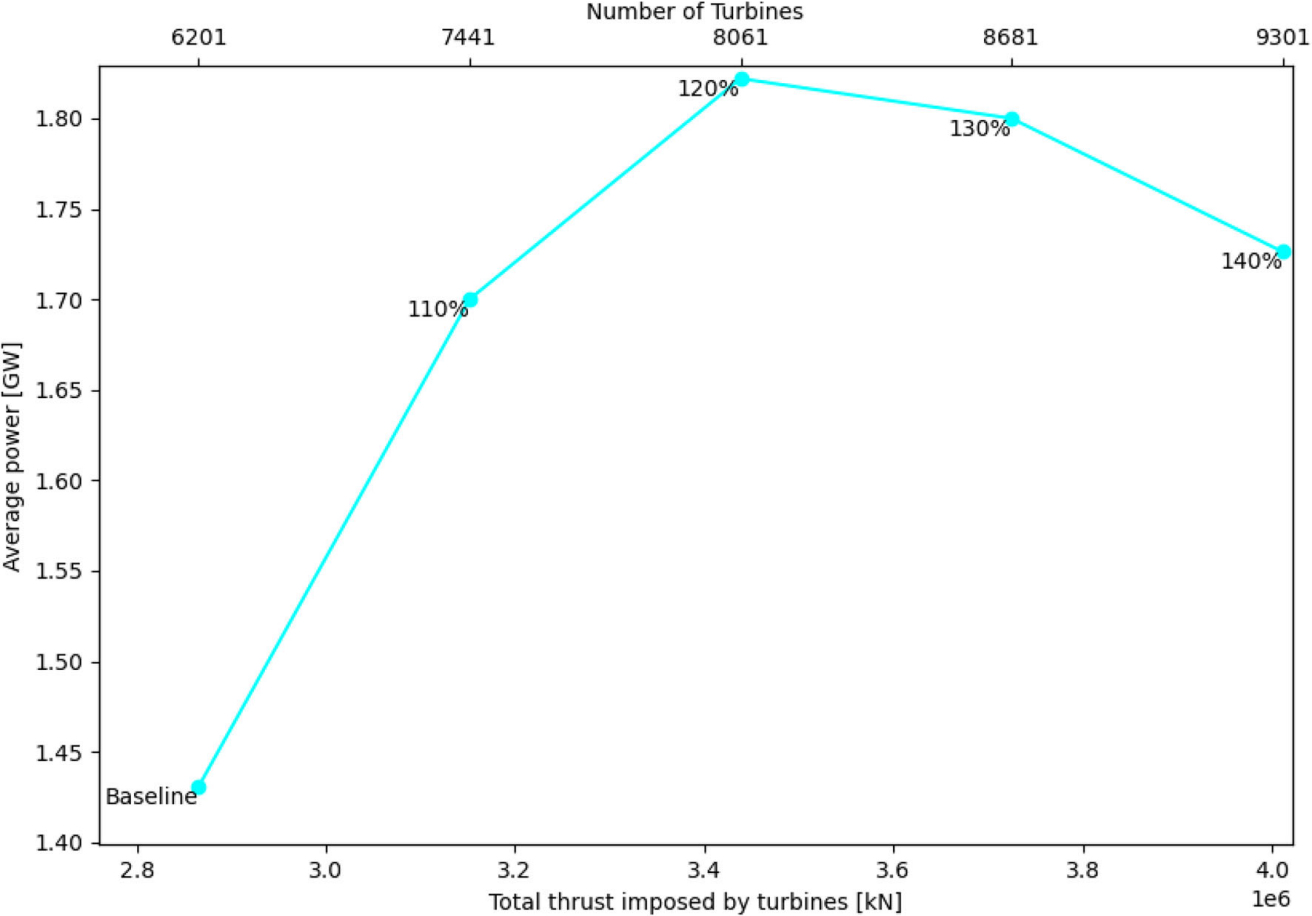
Average power output as turbine density increases across all five arrays. The figure shows the relationship between power generation and the thrust imposed on the flow as turbine density is incrementally increased by up to 40%

The five arrays case is taken as the baseline case, where the turbine density for each array area is based on a 0.16 blockage as in Patel et al. (2024). The turbine density across each array is increased by 10% until 40% more turbines are deployed evenly across the arrays. The average power is plotted against the number of turbines and thrust imposed by the turbines in Fig. 14.

The average power increases as more turbines are deployed, reaching a peak with 8000 turbines (20% increase), resulting in a power output of 1.82 GW. However, beyond this threshold, further increasing the turbine density leads to a decrease in power because of the significant thrust on the flow causing a significant decrease in velocity.

It is important to consider the impact of the increasing thrust has on the flow as the number of turbines increases. Table 5 presents the maximum difference in M$_2$ current amplitude and phase for each case of increasing turbine density compared to the ambient case with no turbines. The baseline case of five arrays results in an maximum 0.97 m/s change in M2 current amplitude and 3.05$^{\circ }$ change in phase. The change in current amplitude reaches a maximum of 1.49 m/s in the 140% case and exceeds 1 m/s in all cases with increased turbine density. This highlights the complexity of balancing maximising power yield without significantly affecting the natural environment of the site, and the maximum change in M$_2$ current indicates how the flow is being impacted. The preservation of the marine environment will always be a priority, therefore, significant changes in velocity across the site are unfavourable. It is unlikely that the maximum power yielded by the 120% turbine density case would be developed because of its potential negative impact on the natural environment.

**Table 5:** Change in M$_2$ current amplitude and phase for the five arrays case with increasing turbine density compared to the ambient case with no turbines

Turbine density	Change in M$_2$ current amplitude [m/s]	Change in M$_2$ current phase [$^{\circ }$]
Baseline	0.97	3.05
110%	1.33	5.92
120%	1.46	7.83
130%	1.45	6.59
140%	1.49	11.37

**Fig. 15:**
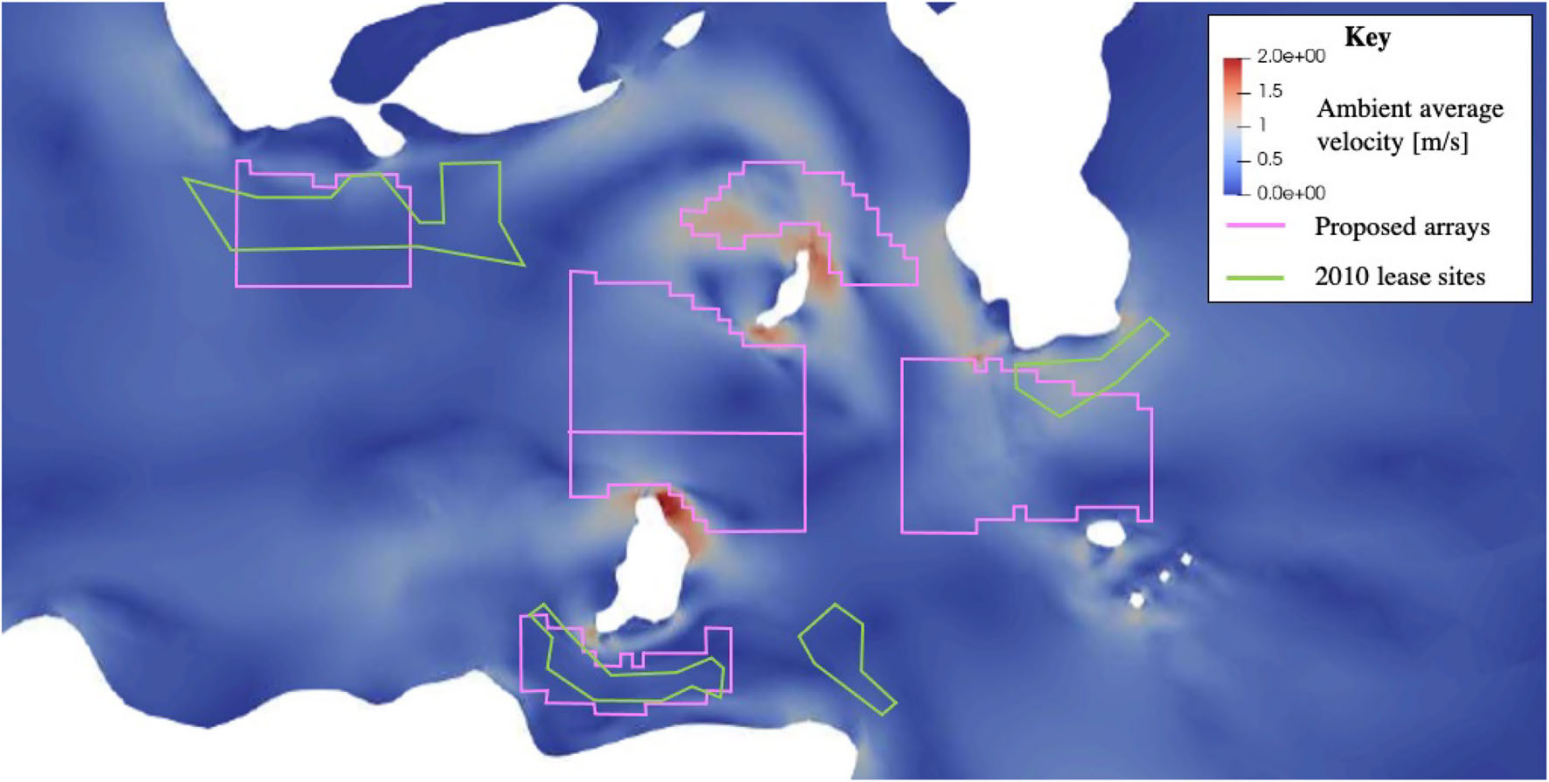
Time-averaged flow velocity for the ambient case (no turbines) with the Crown Estates 2010 lease sites and the proposed arrays in this study illustrated

### Implications for the Pentland Firth resource

The results from this study demonstrate the range of the practical resource in the Pentland Firth based on different development strategies and priorities. The maximum power yielded from the 5 arrays in this study is 1.82 GW (120% density case). However, if the change in M$_2$ current amplitude is to be within 1 m/s from the ambient case, the resource is restricted to 1.43 GW. In the case of shipping remaining a priority for seabed usage, the restriction of a navigation channel results in a 25% reduction in power output relative to not having this restriction and a minimal change in capacity factor.

Figure 15 presents the time-averaged flow velocity for an ambient case with the arrays defined and proposed in this study, through the extension of the heterogeneous framework, and the original 2010 lease sites. Two of the arrays defined in this study are closely aligned to the original lease sites. The original lease sites are more conservative in their occupation of the seabed, however, they are missing key areas of the resource and were not defined with the consideration of the impact on the flow as the site is developed. The arrays proposed in this study have utilised the specification of rated speed across the site to define arrays sequentially and the interaction between arrays is accounted for in their definition, therefore, maximising extraction of the resource.

In comparison with other resource assessments conducted in the Pentland Firth, the range of the resource quantified in this study is in the same order of magnitude as O’Hara Murray and Gallego (2017); De Dominicis et al. (2018) and within the Adcock et al. (2013) upper bound assessment. It is noted that in this study, power capping, maintenance periods, generating losses, etc are not accounted for. Roughly accounting for these factors it is reasonable to say that it is unlikely that average power generated from the Pentland Firth would exceed 1 GW. However, the primary purpose of this study is to look at how the Pentland Firth could be developed taking into account as many constraints as is reasonable in a single paper—whilst the study can feed into the literature on the Pentland Firth resource, it does not set out to find a definitive number for the resource.

This paper has explored one approach to developing the Pentland Firth as a tidal stream site. To do this, significant assumptions about what is feasible in terms of development have been made, based on the literature, which can be challenged. However, given the complexity of the problem, setting out one possible scenario for how the Pentland Firth would be developed and exploring what is predicted to happen provides valuable insight. A key limitation of the present work is that economic factors have not been considered, to keep the scale of the study tractable and also because the uncertainty of costs would likely to overwhelm other considerations and factors.

## Conclusion

By considering practical development scenarios in the Pentland Firth, this study demonstrates that interactions between tidal stream arrays are present but generally moderate. Across the development scenarios examined, the average power output of an individual array is unlikely to change by more than approximately 20% due to array interactions. This finding suggests, although not optimal, it is reasonable to design and develop arrays in isolation provided turbine thrust levels are not excessive. To note, if higher thrust turbines were used in this study (or turbines with larger support structures) then the interactions would be greater. This reinforces the view that sites should be leased based on the allowable thrust rather than on the basis of power. Considering thrust would help minimise both environmental impact and interaction between sites.

The results further demonstrates the interaction effects dependent on the relative positioning of arrays. Arrays broadly arranged in parallel with the dominant flow can experience positive interactions, leading to modest increases in power output, whereas arrays positioned in series tend to exhibit negative interference and reduced power generation.

Incorporating the practical constraint of a shipping navigation channel and restricting development reduces the total average power output by approximately 25% compared to an unconstrained scenario, whilst largely preserving capacity factors. The redistribution of flow around the modified array configuration reduces both positive and negative interaction effects, further highlighting the sensitivity of array interactions to spatial deployment and blockage.

Increasing turbine density across the arrays highlights the limit to resource exploitation. While average power output increases with turbine deployment up to a peak of approximately 1.82 GW at a 20% increase in turbine density relative to the baseline case, further increases in turbine density results in reduced power due to excessive thrust. When environmental constraints are imposed, specifically limiting changes in M$_2$ current amplitude to within 1 m/s of ambient conditions, the practically exploitable resource is reduced to approximately 1.43 GW.

Overall, this study suggests that whilst the Pentland Firth represents a substantial tidal stream resource, realistic development constraints and flow-array interactions are likely to limit average power generation to around 1 GW, consistent with previous upper-bound estimates. The work provides a structured framework for understanding how large-scale tidal stream developments may be considered whilst balancing energy yield and constraints on the resource.

## Data Availability

No datasets were generated or analysed during the current study.
